# Characteristics of Dental Services in Rural, Suburban, and Urban Areas Upon the Implementation of Indonesia National Health Insurance

**DOI:** 10.3389/fpubh.2020.00138

**Published:** 2020-05-12

**Authors:** Iwan Dewanto, Sittichai Koontongkaew, Niken Widyanti

**Affiliations:** ^1^Faculty of Medical and Health Science, School of Dentistry, University Muhammadiyah Yogyakarta, Bantul, Indonesia; ^2^Faculty Dentistry, Thammasat University Thailand, Bangkok, Thailand

**Keywords:** dental disease pattern, dental therapy pattern, community health centers, rural, suburban, urban

## Abstract

**Introduction:** The implementation of Indonesia National Health Insurance (NHI) for oral health needs to be evaluated by observing the dental disease patterns and dental therapy patterns from community health centers (CHCs) in the rural area, suburban area, and urban area. The aim of the study is to describe the characteristics of dental services in rural, suburban, and urban areas after the implementation of NHI on CHCs in the Special Region of Yogyakarta in 2014.

**Materials and methods:** This is an observational study with a cross-sectional research design. The study used quantitative data obtained from dental records at selected CHCs. Using a purposive sampling method, 30 CHCs as unit analysis were collected from rural, suburban, and urban areas. The data were collected from January 2014 to December 2014.

**Results:** Data from 26,554 patients were collected from dental records of dental clinics at CHCs. There were 5829 patient dental records from rural areas, 12,327 from suburban areas, and 8938 from urban areas. The primary dentist tends to provide services without clinical intervention on periodontal problems, abscesses, and lesions. Clinical interventions were mostly provided for prolonged retention and deposits on teeth. Primary dentists in suburban areas tend to provide clinical intervention on caries disease compared to those in rural and urban areas. Statistically significant differences (*p* < 0.05) were observed among locations in the pattern of providing clinical interventions on caries, abscess, lesion, prolonged retention, deposits on teeth, and other problems. No difference was recorded only on periodontal disease.

**Discussion:** This study found that each area has different characteristics of dental disease and dental therapy patterns. Each area has a significant difference in the pattern of the clinical intervention of dental disease except in periodontal problems.

## Introduction

In 2014, the Indonesian government launched the National Health Insurance (NHI) system, which includes dental services. This policy was created as a result of the resolution passed by the World Health Assembly, which urged countries to develop health financing systems that would allow all people to have access to the required services (1). Since the implementation of this policy, the primary dentists surveyed have been worried that the number of patients receiving therapy would increase and that their workloads would increase accordingly (2). Therefore, it is necessary to evaluate the implementation of the NHI system for oral health by observing disease patterns and the dental therapy treatments carried out. In addition, there are no available data for oral disease pattern that meets the NHI criteria (3).

In Indonesia, community health centers (CHCs) play an important role in the NHI program by delivering comprehensive and integrated health services to the community under the authorization of the district health office. Indonesian CHCs have been the subject of studies on the implementation of the national program through required monitoring and reporting, which extends to dentists as providers of public services. Information and characteristics on dental diseases and therapies can be used by the district health office to improve their performance. The changes in dental disease patterns in the population reflect systematic efforts made by dental health services regarding preventive and curative dental care (4). Owing to their limited resources, many developing countries can only provide tooth extraction services to relieve pain and tooth problems, leaving millions of people suffering from tooth loss (5). Dental caries and periodontal disease are a considerable public health problem in the majority of developing countries. Significant disparities within and between regions have been observed for epidemiologic indicators of oral disease. The prevalence of tooth loss rates and experience of oral problems vary substantially by World Health Organization region and national income (6). However, the lack of adequate data in most countries makes the prediction of future oral health and manpower needs a precarious procedure. This indicates the urgent need for regular monitoring of oral health status and dental services in all countries.

Decision makers must have reliable information and the capacity to assess health needs, choose intervention strategies, design appropriate policy options for local or national circumstances, monitor performance, and manage changes. The World Health Organization has strengthened the surveillance of non-communicable diseases at global, regional, and national levels over recent years. This has been done for several reasons. Firstly, surveillance offers a systematic approach to data collection and helps countries monitor and evaluate emerging disease patterns and trends. Secondly, the government can formulate policies and programs to prevent disease and to measure the progress, impact, and efficacy of preventive efforts already in operation. Thirdly, surveillance systems may help strengthen health care provision and supply evidence for introducing procedures, programs, and policies (7).

Unfortunately, dental services either receive minor attention or are ignored altogether. A behavior model of the dental care process should capture different reasons for the dental visit. In a given population, individuals can be divided into two groups, namely, symptomatic and asymptomatic. Both groups may choose to visit or avoid visiting the dentist. The former group chooses to visit primarily to eliminate symptom(s), while asymptomatic patients visit the dentist to prevent future problems. If signs of disease are detected during an oral examination (e.g., bleeding gums), asymptomatic patients may seek therapy to prevent minor problems from becoming major ones in the future (8).

In the developing and developed world, many rural individuals must travel substantial distances to obtain primary medical care, requiring significantly longer travel times to reach care than their urban counterparts (9). Furthermore, some rural areas have a higher proportion of uninsured and individually insured residents than urban areas (10). Despite negative health behaviors, many aspects of rural social life contribute to positive health outcomes. Rural areas frequently have more advantages in denser social networks, longer duration of social ties, shared life experiences, high quality of life, and norms of self-help, and reciprocity (11).

In Indonesia, individuals with the same needs for dental care who have different abilities to pay for that care may not receive equal dental therapy. The perceived need for utilization of dental care was found to be low among Indonesians; moreover, the rate of unmet dental care needs is relatively high (12). Patient behavior can lead to and affect dental visit rates and disease pattern. Patient behavior regarding dental disease and therapy can be divided into rural, suburban, and urban patterns. Three factors affecting individuals' health care-seeking behaviors are predisposing factors (sociodemographic status, education, attitudes, beliefs about health, etc.), enabling factors (income, health insurance, etc.), and illness level. Thus, the utilization of dental services is related to income, dental insurance, oral health indicators, level of education, age, gender, and supply of dentists per capita. The other influential aspects are societal, environmental (living and working conditions), structural (structure and function of the dental health service system), and psychological factors (13). There are several challenges to the delivery of oral health care to rural populations, such as a lack of manpower, poor accessibility, and unaffordability. Therefore, community outreach programs should be tailored to the needs of the local population (14).

Indonesia is a developing country with a large population, which is the fourth most populous country. The Basic Health Survey conducted by the Indonesia Ministry of Health (15) showed that almost all provinces in Indonesia had significantly increased prevalence of active caries. Overall, the active caries prevalence was 43.4% in the year 2007 (16) and had reached 53.2% in the year 2013. Based on the World Oral Health report, developed and developing countries need to encourage primary health care models for providing essential oral health care (17). However, the uneven distribution of dentists in Indonesia, who are mostly concentrated on the Java and Sumatra Islands, have led to unequal patterns in the provision of oral health services (18).

In ecological research, the urban–rural gradient is described via various indicators, which can be classified into three basic groups: (1) demographic variables; (2) physical variables such as density of roads or buildings, percentage of urban land cover, or distance from an urban center; and (3) landscape metrics such as the mean size or fractal dimension of patches of land (19). Coombes and Raybould (20), argued, however, that in an increasingly complex pattern of settlement linked by socioeconomic polarization, no single measure can represent all of the distinct aspects of settlement structure, which is of interest to public policy. They suggested that there are at least three key dimensions to modern human settlement patterns, which are quite distinct from each other and should all be taken into account by policymakers when allocating resources or designing programs. These dimensions use criteria such as overall population, population density, commuting patterns, and/or distance from other settlements to determine if a settlement is urban, suburban, or rural. Suburban can be defined as an area far from the city core but close enough for commuting (21). It functions as a place of residence for people who work in the main city and factory workers in satellite cities.

The Special Region of Yogyakarta, Indonesia, consists of four districts and one municipality with a high population density. In fact, this density problem in Yogyakarta Province reflects a major problem in all of Indonesia, which is the unequal distribution of the population between rural and urban areas. Based on a report by Central Bureau (22), public facilities in Yogyakarta are considered the best on Java Island in terms of the quality of education, health care, and welfare. However, it is noteworthy that there is an economic discrepancy between the rich and the poor. Thus, the prevalence and severity of dental caries is higher in areas with higher income inequality (23). A national health survey found that the decay missing filling index score for the Special Region of Yogyakarta is 5.9 at 12 years of age, meaning that the province ranks third highest for index of decay missing filling in Indonesia (15). Thus, there is a need to explain the discrepancy in the Special Region of Yogyakarta between the presence of a sufficient number of dentists and poor outcomes in the effort to decrease active caries.

In addition, there are no available data for oral disease patterns and dental therapy pattern in this region that meet the NHI's standards for tracking changes in the dental service characteristics. Therefore, the aim of the study is to describe the characteristics of dental services in rural, suburban, and urban areas after the implementation of NHI on CHCs in the Special Region of Yogyakarta in 2014.

## Materials and Methods

This is an observational descriptive study with a cross-sectional research design. The study used quantitative data obtained from dental records of NHI participants who have visited at CHCs in four regions and one municipality in the Special Region of Yogyakarta. All of the CHCs in the Special Region of Yogyakarta have a dental clinic. The selected CHCs' dental clinics were determined based on similar demographic and condition in several CHCs in each area. The population density appears in Yogyakarta Municipality and Sleman Regency, which has the highest density despite its small area; thus, it can be classified as an urban area. Meanwhile, the Bantul and Kulonprogo Regencies are located far from city centers and are labeled as suburban areas. The Gunung Kidul Regency, which is far away from city centers and has the lowest density despite its large area, is labeled as a rural area.

The urban areas have 43 CHCs, found in Yogyakarta Municipality with 18 CHCs and Sleman Regency with 25 CHCs. The suburban areas have 48 CHCs, found in Bantul Regency with 27 CHCs and Kulonprogo Regency with 21 CHCs, and rural areas (Gunung Kidul Regency) have 30 CHCs. Thus, the total number of CHCs at the Special Region of Yogyakarta is 121 CHCs.

Purposive sampling was used to define 30 CHCs from 121 CHCs as a unit analysis for this research. The selection of CHCs for each area was conducted using a purposive sampling method based on inclusion criteria. The inclusion criteria for selected dental clinics were having a cooperation agreement with Badan Pengelola Jaminan Sosial (Social Health Insurance Administration Organization) for at least 1 year, willing to cooperate, having complete dental equipment in accordance to standard dental package equipment guidelines from the Health Ministry, and having a good administration for collecting data. Dental clinics at CHCs that did not meet the above criteria were excluded.

The surveyor collected data from dental records at the clinics. These data were used to describe the implementation of CHC dental services from January 2014 until December 2014 in CHCs in rural, suburban, and urban areas. The observation of dental records was conducted to determine two domain patterns:

1. The dental disease patterns are the data that will give a brief description of the specific pattern for dental disease in each area (urban, suburban, and rural areas). The dental diseases are defined from Indonesia Health Ministry regulation No. 62 imposed in 2015 about practical guidelines for primary dentists in Indonesia. There are 26 diseases in the practical guidelines for Indonesia's primary dentists to be implemented by the Indonesian National Health Insurance (NHI).
2. Dental therapy pattern. These are the therapy data that have been delivered by the dentists for each dental disease. Dental therapy pattern will be defined from Indonesia Health Ministry regulation No. 62 imposed in 2015 about practical guidelines for primary dentists in Indonesia. There are eight dental therapies in the practical guidelines for Indonesia's primary dentists to be implemented for Indonesian NHI.

Calibration was conducted to minimize errors created by the observer or during transcription and notation of the medical records. Because the Indonesia Ministry of Health's regulation No. 62 was established after the NHI was implemented, it is possible that some of the diagnoses and dental therapies may have been written differently by primary dentists at CHCs. Based on this condition, the observers had to be dentists in order to interpret the diagnoses and dental therapies found in the medical records. This study involved four main observers, who graduated from the School of Dentistry, University Muhammadiyah Yogyakarta at least 1 year prior to the study. Through an interactive calibration session, they were trained to transcribe a medical record into a standard code and to enter it into the software that was developed for this study. The typical codes for diagnoses and dental therapies were based on the International Classification of Diseases 10 and International Classification of Therapy 9 (with clinical modification) as per the Indonesia Ministry of Health's regulation No. 62. If there are different diagnoses and if dental therapy has been written differently by the primary dentist at CHCs, a new code will be created since it is not in agreement with the code of the Indonesia Health Ministry regulation No. 62 imposed in the year 2015.

This research has been reviewed and approved by the Ethics Committee of the Medical Faculty and Health Science of Muhammadiyah University Yogyakarta. All selected CHCs used in this study have obtained permits through the appropriate bureaucratic mechanism in their respective regions. SPSS 25 has been used for statistical analysis, and the interdependence of characteristics was evaluated by chi-square (χ^2^).

## Results

Data were collected from a total of 26,554 patient dental records at CHCs' dental services in the Special Region of Yogyakarta. The data were derived from files of patients' dental records as a participant of NHI who has visited CHCs' dental services in 2014. There were 5,829 patient dental records from rural areas, 12,327 from suburban areas, and 8,938 from urban areas. Suburban areas had the highest patient visit rates for dental services, while the lowest visit rates came from rural areas. Patients' demographic data, such as economic status (based on the patient's job description), gender, and age were also recorded. Unfortunately, socioeconomic data could not be presented here because not all primary dentists filled the patients' job descriptions at dental records. In fact, it was found that several primary dentists at CHCs did not collect any socioeconomic background on their patient records. Patient age categories were based on the Indonesian Ministry of Health classification. The age group with the highest number of visits was adults ages 26–35 years (*n* = 8107), followed by children ages 0–16 years (*n* = 6844), and older adults ages 46–65 years (*n* = 6233).

**Figure 1:**
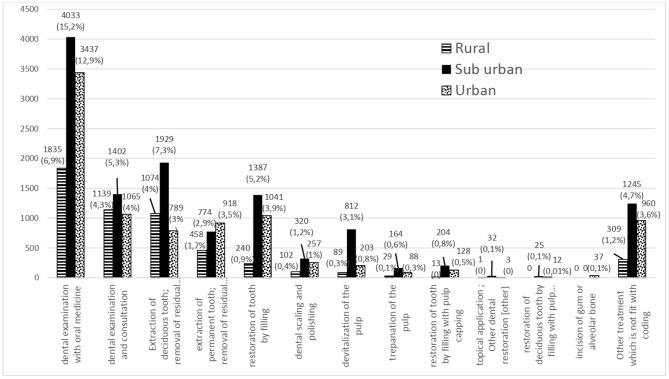
Dental disease patterns in urban, suburban, and rural areas of the Special Region of Yogyakarta in 2014.

The main dental diseases found in rural areas were necrosis of the pulp, retained root, and periodontal disease problems. This disease pattern result reveals differences in the dental pattern among patients in rural, suburban, and urban areas (Figure 1). In addition to the number of cases, Figure 1 also presents a percentage, which is obtained based on the calculation of the percentage of all existing cases. In rural areas, a retained (persistent) primary tooth (2,436 cases, 9.91%) and necrosis of the pulp (1,419 cases, 5.77%) were the major diseases found. These major diseases were followed by a retained dental root (permanent) (462 cases, 1.88%) and aggressive periodontitis/periodontal abscess (432 cases, 1.76%). However, dental disease patterns were different in suburban areas. There, the most prevalent disease was necrosis of the pulp (3,542 cases, 14.41%) followed by a retained (persistent) primary tooth (2,436 cases, 9.91%). There was a higher prevalence of aggressive periodontitis/periodontal abscess (1,458 cases, 5.93%) in suburban compared to rural areas. Irreversible pulpitis (791 cases, 3.22%) was also high among suburban patients. In urban areas, the disease with the highest prevalence was necrosis of the pulp (2,078 cases, 8.45%) followed by aggressive periodontitis/periodontal abscess (1,106 cases, 4.5%). In urban areas, the number of dental caries penetrating into dentin was higher (987 cases, 4%) than that in rural areas (162 cases, 0.66%) and suburban areas (558 cases, 2.27%).

In this study, 15 diagnoses of dental diseases were found that did not fit the 26 codes provided by the Indonesia Ministry of Health regulation No. 62. This indicates that there are several dental diseases that are not covered by the Indonesian NHI scheme. Unfortunately, these diseases were related to common dental problems in Indonesia, such as third molar impaction (716 cases, 2.16%). Third molar impaction was prevalent in all areas, with the highest prevalence in urban areas. The 15 diagnoses that do not fit with the coding are as follows: third molar impaction, abscess with trismus, malocclusion, post-extraction infection/dry socket, carcinoma, tumor, xerostomia, mucocele, exfoliation of teeth due to systemic causes, radicular cyst, broken bridge, broken partial denture, epulis, rudimentary teeth at palate, and unspecified dental disease (such as orofacial pain). There were 9 types of diseases found in rural areas, 14 in suburban areas, and 3 in urban areas that did not fit the standard codes. This indicates that the suburban areas have a greater variety of dental diseases than the other two areas. It is noteworthy that all three areas shared three dental diseases that did not fit the standard codes, namely, third molar impaction, post-extraction infection/dry socket, and malocclusion.

**Figure 2:**
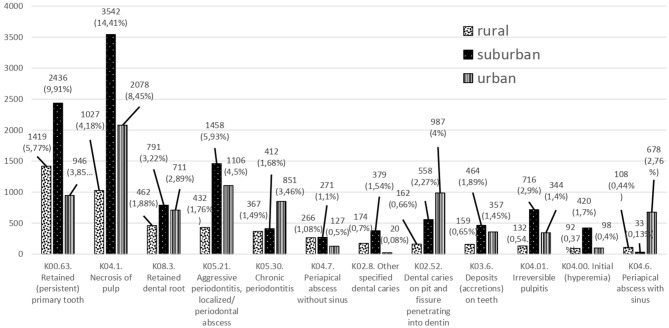
Dental therapy patterns in urban, suburban, and rural areas of the Special Region of Yogyakarta in 2014.

Dental examinations were the most common dental therapy delivered in CHCs in the Special Region of Yogyakarta (Figure 2). In addition to the number of therapies, Figure 2 also presents a percentage, which is obtained based on the calculation of the percentage of all existing therapies. In rural areas, dental examinations with oral medicine therapy (1,835 cases, 6.9%) or with consultations (1,139 cases, 4.3%) were the most common therapies. The extraction of deciduous teeth was more common than permanent teeth. This trend is in line with the disease pattern and visit rate by age. Filling and dental scaling therapies were not as common as extraction therapies. In the Special Region of Yogyakarta, premedication therapy was the preferred choice of service delivery among primary dentists.

The differences in dental therapy patterns are indicative of various dental conditions in the Special Region of Yogyakarta. A total of 2,514 therapies (10.23%) were found that did not fit the Ministry of Health's standard codes. This indicates differences in the therapy approaches among primary dentists in the region.

There were seven types of dental therapies in rural areas that did not fit standard codes, eight in suburban areas, and six in urban areas. Suburban areas had the highest provision of dental therapy (1,245 cases, 4.7%) compared to rural (309 cases, 1.2%) and urban (960 cases, 3.6%) areas. Interestingly, the most favored therapy for solving irreversible pulpitis and initial hyperemia was lesion sterilization by tissue repair (3 Mix MP). This treatment was most prevalent in suburban areas, and the use of 38 endodontic therapies was also shown in urban areas, which is not found in the other two areas.

There were clear differences in the number of dental health facilities between rural, suburban, and urban areas. Urban areas had the highest number of referrals (532 referrals), which was not in line with the disease pattern data. It seems that the availability of many optional dental health facilities in the urban area led to urban dentists referring cases to other facilities.

The resulting data from dental diagnoses and dental therapies could be presented and analyzed with chi-square as category data. The diagnosis data have been categorized as caries disease, periodontal diseases, abscess, lesion, prolonged retention, deposits on teeth, and other problems. Dental therapies could be categorized into clinical intervention and without clinical intervention (premedication, consultation, and referral). Characteristics of the clinical intervention pattern are shown in Table 1. The results of the data show that, in percentage, primary care dentists tend to provide services without clinical intervention on periodontal problems, abscesses, and lesions. Clinical interventions were mostly provided for prolonged retention and deposits on teeth. What is interesting in these data is that dentists in suburban areas tend to provide clinical intervention on caries disease compared to those in rural and urban areas. Statistically significant differences (*p* < 0.05) were observed among locations in the pattern of providing clinical interventions on caries, abscess, lesion, prolonged retention, deposits on teeth, and other problems. No difference was recorded only on periodontal disease.

**Table 1:** Characteristics of the clinical intervention pattern in percentage.

	**Rural**		**Suburban**		**Urban**		**Chi-square**
	**With clinical intervention**	**Without clinical intervention**	**With clinical intervention**	**Without clinical intervention**	**With clinical intervention**	**Without clinical intervention**	
Caries	778 (37.29%)	1,308 (62.7%)	3,196 (58.81%)	2,238 (41.18%)	1,563 (47.69%)	1,714 (52.3%)	0.008^*^
Periodontal	288 (25.44%)	844 (74.56%)	806 (35.43%)	1,469 (64.57%)	831 (39.97%)	1,248 (60.03%)	0.72
Abscess	16 (3.86%)	398 (96.14%)	0 (0)	546 (100%)	157 (19.6%)	644 (80.39%)	0.00^*^
Lesion	5 (6.02%)	78(93.98%)	10 (7.09%)	131 (92.91%)	14 (27.45%)	37 (72.55%)	0.00^*^
Prolong retention	1,056 (72.73%)	396 (27.27%)	1,799 (80.31%)	441 (19.69%)	689 (63.68%)	393 (36.32%)	0.04^*^
Deposits on teeth	91 (53.22%)	80 (46.78%)	317 (68.76%)	144 (31.24%)	266 (74.51%)	91 (25.49%)	0.03^*^
Other problem	205 (43.99%)	261 (56.01%)	791 (51.4%)	748 (48.6%)	406 (31.47%)	884 (68.53%)	0.015^*^

* *Statistically significant differences*.

## Discussion

Past research has documented the differences between urban and rural health care, usually expressed in terms of health care access, utilization, and services (24). By utilizing a framework to examine determinants of health, researchers can identify environment-specific factors that may contribute to different health outcomes for urban, suburban, and rural residents (25). A common disease trend that emerged in all areas is necrosis of the pulp. Ideally, necrosis of the pulp has two optional treatments: extraction or root canal treatment if the crown is still suitable. These two optional treatments have their own advantages and disadvantages. If residents choose the extraction treatment, they will lose their teeth, and furthermore, they will need dentures. If they choose the root canal treatment, it will be costlier and more time-consuming. Repeated visitation is required for root canal treatment, and this will increase the rate of visit and cost expenditure; this circumstance is not convenient for the patient and not suitable for the primary dentist. Unfortunately, the second choice is not included in the benefit package program; on the other hand, there is an evaluation indicator report for the Health Ministry regarding the ratio of extraction compared with filling treatment.

The major problems of oral health at CHCs are necrosis of the pulp and periodontal problem; this could present as a risk factor for dentists who have implemented the NHI with capitation funding scheme. This result is a factor that makes the implementation of the NHI difficult. This finding is in line with the result of primary dentists who perceive capitation revenue as not suitable for carrying out dental services. This result adds to the anxiety experienced by dentists due to the implementation of the NHI (2). The disease pattern result provides an argument for the primary dentists in that there will be a large expenditure involved in the implementation of the NHI.

In the dental therapy pattern, it is obvious that the implementation of the NHI for oral health in Indonesia has a policy problem, which is the determination of eight dental treatments as part of the benefit package and the amount of the capitation, wherein primary dentists face problems in terms of dental diseases that are not covered by the NHI. The primary dentist seems to choose the symptomatic treatment to delay the problem rather than solving the problem. This could be due to the fact that premedication treatment at CHCs does not incur any cost to deliver this treatment. As a public service, drugs should be provided by the local government and distributed to each CHC monthly. Hence, the primary dentist does not have any drug expenditure for this dental treatment that could be used. This is the reason why premedication is the primary dental therapy pattern.

In the implementation of the NHI, the primary dentist should be the “gatekeeper”; this scheme was written in the “Practical Guide of Gatekeeper Concept Health Facilities BPJS Health.” As a gatekeeper at the first level of health services, the primary dentist should have four functions: being the first contact, continuity, comprehensive health care, and coordination. It seems that due to many optional dental health facilities in urban areas, the urban dentist tends to refer several cases to other dental health facilities. This condition exposes primary dentists in urban areas not implementing the gatekeeper function, especially to deliver continuity, comprehensive health care, and coordination. This finding was similar with that of the study of Budiarto and Oktarina (26), which stated that the readiness of dentist practice in Central Java as a gatekeeper is 50% continuity, 37.5% comprehensive health care, and 50% coordination.

Each area shared a major dental problem, which is necrosis of the pulp. However, the dental therapies provided were primarily symptomatic therapies (premedication). This indicates that primary dentists only delay dental problems rather than solving them. This was shown by the prevalence of premedication and consultation as the main therapies provided in all areas. Patients seeking care for emergency dental issues often received temporary therapy through symptomatic relief (antibiotics and narcotics), which does not definitively treat the underlying disease process (27).

Indonesia's NHI has increased the probability of individuals seeking outpatient and inpatient care. This impact is stronger in suburban areas, which likely comes from self-employed individuals or people who worked in the informal sector. This finding is consistent with evidence from other countries (28). Based on disease pattern in suburban areas, the primary dentists in this area should take an emergent reaction to give any required dental therapy if there are patients going to the CHCs.

Characteristics of the clinical intervention pattern show that the primary care dentist tends to provide services without clinical intervention on periodontal problems, abscesses, and lesions. Primary dentists at the Special Region of Yogyakarta mostly provided clinical intervention for prolonged retention and deposits on teeth. The extraction of the deciduous teeth was the most preferred therapy, which is in line with the disease pattern. This may be because extraction of the deciduous teeth is a therapy that does not require complex equipment and materials. It seems that the primary dentists in the Special Region of Yogyakarta choose convenient dental therapies that do not require complex equipment or materials.

Interestingly, primary dentists in suburban areas tend to provide clinical intervention on caries disease compared to those in rural and urban areas. The suburban area exhibits a transitional change of disease pattern. There is a change of sequence pattern for irreversible pulpitis disease and dental caries on pit-fissure penetrating into dentin disease in suburban areas. Primary dentists in suburban areas deliver the LSTR (the lesion sterilization by tissue repair) treatment higher than other areas, and endodontic treatment is provided, which is not provided in other areas. This circumstance indicates that the suburban dentist tends to deliver the dental treatment compared with other areas. In the pattern of providing clinical interventions on caries, abscess, lesion, prolonged retention, deposits on teeth, and other problems in each area, there were no differences recorded. The similarity among each area in terms of clinical intervention approach was only found for periodontal disease problems.

Suburban areas have a higher rate of patient visits with various types of dental diseases and urban areas have a moderate visit rate and better patient awareness of dental health; thus, networks should be created between dental facilities to maintain dental services. Finally, this study revealed a lack of preventive or promotive efforts to reduce oral health problems through comprehensive dental care.

This study found that each area has different characteristics of dental disease and dental therapy patterns. Each area has a significant difference in the pattern of clinical intervention of dental disease except in periodontal problems.

## Data Availability Statement

The data analyzed in this study were obtained from dental records at CHCs in the Special Region Yogyakarta Province, Indonesia. However, restrictions apply to the availability of these data and therefore they are not publicly available. Nonetheless, data are available from the authors upon reasonable request.

## Ethics Statement

This research has been reviewed and approved by the Ethics Committee of the Medical Faculty and Health Science of Muhammadiyah University Yogyakarta.

## Author Contributions

ID wrote the main text, executed the study, collected data, and drafted and developed the text. SK was responsible for the design and for providing guidance, direction, correction, and constructive criticism, and sharing his valuable knowledge and experience. NW provided academic advice and interpreted the findings. All authors read and approved the final manuscript.

## Conflict of Interest

The authors declare that the research was conducted in the absence of any commercial or financial relationships that could be construed as a potential conflict of interest.

## Abbreviations

NHI: National Health Insurance
CHC: Community health center.
